# Fundamental roles of the innate-like repertoire of natural antibodies in immune homeostasis

**DOI:** 10.3389/fimmu.2013.00004

**Published:** 2013-02-05

**Authors:** Jaya Vas, Caroline Grönwall, Gregg J. Silverman

**Affiliations:** ^1^Laboratory of B Cell Immunobiology, Department of Medicine, New York University School of MedicineNew York, NY, USA; ^2^Laboratory of B Cell Immunobiology, Department of Pathology, New York University School of MedicineNew York, NY, USA

**Keywords:** immunoregulation, innate-like, natural antibody

## Abstract

The composition of the early immune repertoire is biased with prominent expression of spontaneously arising B cell clones that produce IgM with recurrent and often autoreactive binding specificities. Amongst these naturally arising antibodies (NAbs) are IgM antibodies that specifically recognized amaged and senescent cells, often via oxidation-associated neo-determinants. These NAbs are present from birth and can be further boosted by apoptotic cell challenge. Recent studies have shown that IgM NAb to apoptotic cells can enhance phagocytic clearance, as well as suppress proinflammatory responses induced via Toll-like receptors, and block pathogenic IgG-immune complex (IC)-mediated inflammatory responses. Specific antibody effector functions appear to be involved, as these anti-inflammatory properties are dependent on IgM-mediated recruitment of the early recognition factors of complement. Clinical surveys have suggested that anti-apoptotic cell (AC) IgM NAbs may modulate disease activity in some patients with autoimmune disease. In mechanistic studies, anti-AC NAbs were shown to act in dendritic cells by inhibition of the mitogen-activated protein kinase (MAPK) pathway, a primary signal transduction pathway that controls inflammatory responses. This immunomodulatory pathway has an absolute requirement for the induction of MAPK phosphatase-1. Taken together, recent studies have elucidated the novel properties of a class of protective NAbs, which may directly blunt inflammatory responses through a primitive pathway for regulation of the innate immune system.

## INTRODUCTION

The evolutionary emergence of the combinatorial antigen receptor system of variable region (V) gene rearrangements in lymphocytes has provided a greatly enhanced capacity for specific recognition of an immense range of ligands. In humans, the antibody system can generate B cell antigen receptors (BCR) encoded by more than 10^20^ genetically distinct variable region rearrangements. Considering that each individual has only about 10^11^ lymphocytes, if the primary B cell repertoire was indeed generated randomly, there would belittle or no recurrence in the antibody gene sequences in the somatically generated repertoires of the billions of humans on the planet. In the following sections, we review evidence the B cell compartment arises during development with a restricted and biased repertoire, and that the antibody products of these B cell clones may serve to protect the host from both external threats and for the maintenance of internal homeostasis.

## RESTRICTION IN THE EARLY REPERTOIRE

In the mouse, there is a remarkable restriction in the usage patterns of the heavy chain V region (V_H_) genes during early repertoire development (Perlmutter et al., 1985). Surveys of murine antibody sequences have provided extensive evidence of recurrent lymphocyte clones with the same V gene rearrangements in different individuals (Seidl et al., 1997), and emerging data suggests there may be similar patterns in the human B cell repertoire (Jackson et al., 2012). In fact, these biases in the expression of V_H_ rearrangements are first detectable at a time point at which representation cannot be affected by antigenic selection of these IgM-associated clones (Schroeder et al., 1987; Schroeder and Wang, 1990). Moreover, a recent report suggested that the perinatal V_H_ repertoire expressed in human IgA may be even more restricted than the IgM pool (Rogosch et al., 2012). Recurrent biases in the V_H_ expression in the early B cell repertoire have also been reported in other species, such as swine (Sun et al., 1998) and sheep (Jenne et al., 2006), as well as more primitive species, such as the amphibian *Xenopus laevis* (Flajnik and Rumfelt, 2000), and zebrafish (Du Pasquier et al., 2000).

Both humans and mice have circulating IgM antibodies that arise early life without immunogenic challenge and have therefore been termed natural antibodies (NAbs). In fact, neonatal B cells produce IgM antibodies that are readily detectable in the bloodstream at birth, and studies in mice have shown that more than 80% of circulating IgM are produced by a phenotypically distinct mature B cell subset, termed the B-1a cell subset, and characterized by membrane-associated CD5. In general, while some B-1 cells express antigen-receptors for recognition of common bacterial Ags, some B-1 cell clones can also recognize self-antigens, including the phospholipidphosphatidylcholine (PtC), the phospholipid-associated phosphorylcholine (PC) head group, as well as DNA and certain cell membrane proteins (Kantor and Herzenberg, 1993).

B-1 cells are believed to represent a developmentally distinct lineage from their adult counterpart, the bone marrow-derived B-2 subset (reviewed in Hardy, 2006; Baumgarth, 2011). Murine B-1 clones are self-replenishing, which ensures the maintenance of this repertoire, as later in life the capacity for *de novo* generation of mature lymphocytes with the B-1 cell phenotype is limited. Studies by Notkins and colleagues have shown that CD5-bearing human B cells also have a bias toward the production of certain types of autoantibodies (Casali and Notkins, 1989). However, CD5 molecules can represent an activation marker on human B cells, and hence by itself CD5 may not be a rigorous phenotypic marker for this B cell subset in humans (Cong et al., 1991). To address this long standing issue, Rothstein and coworkers have reported a detailed phenotyping scheme, in addition to CD5, for identifying human B cells with the diagnostic features of B-1 cells. The repertoire of these human B-1 cells also appeared to include prominent expression of self-specificities for native DNA and PC-containing antigens (Griffin et al., 2011).

## AUTOREACTIVITY OF B LYMPHOCYTE SUBSETS

In mice, mature B-1 and B-2 lymphocyte subsets can play discrete but complementary functional roles in host defenses (reviewed in Baumgarth, 2011). There are also subpopulations within B-1 cells in addition to CD5^+^ B-1a cells, as B-1b cells (that do not express CD5) make essential contributions to T cell-independent defenses for certain types of infections (Alugupalli and Abraham, 2009). The clonal selection of these distinct B cell subsets may in part reflect differences in their cellular thresholds for negative selection (i.e., BCR-induced cell death) and in their activation requirements for second signals after BCR stimulation. By one estimate, over 70% of BCR-expressing immature B cells in the bone marrow display some level of autoreactivity while the level is much less in recirculating naïve mature B-2 cells (Wardemann et al., 2003). Hence, the immune tolerance checkpoints for B-2 cells that arise from precursors in the bone marrow appear to be generally more stringent in the removal of self-reactivity (i.e., negative selection). In contrast, conserved B-1 cell clonotypes may be positively selected (i.e., enhanced survival and clonal proliferation) by certain types of non-protein self-antigens (Hayakawa et al., 1999), which may include specific types of intracellular antigens (Ferry et al., 2007). As B-1 cells are a major source of circulating IgM in neonates, this may explain why neonatal IgM–NAbs from umbilical cord commonly display features of self-reactivity (Chou et al., 2009).

In the human immune system there is a remarkably strong association between the immune recognition of cell surface *N*-acetyllactosamine/polylactosamine determinants in glycoconjugates and the usage of the V_H_4-34 gene segment (originally termed V_H_4-21; Silberstein et al., 1991). *N*-acetyllactosamine/polylactosamine moieties are common on cell surfaces throughout the body, as these are structural components of the I/i blood group antigens and also constitute the antigenic target of pathogenic autoantibodies in cold-agglutinin disease (Silverman et al., 1990; Silberstein et al., 1991; Grillot-Courvalin et al., 1992; Pascual and Capra, 1992).

The immune recognition of I/i related non-protein antigens may be involved in very different types of immune responses. Using a lectin microarray system, exosomes released by human tumor cell lines were shown to express a shared polylactosamine glycan signature (Batista et al., 2011). These findings extend earlier evidence that I/i related determinants can be preferentially expressed on cells during early development and on their malignant counterparts (i.e., onco-fetal antigens; Feizi, 1988). In addition, V_H_4-34 encoded autoantibodies were found to commonly bind to HIV-1 envelope determinants (Kobie et al., 2012). Batista et al. (2011) have suggested that these glycans reflect a recurrent type of glycan epitope profile on stressed and apoptotic cells (ACs). Taken together, these findings highlight the intertwined nature of immunodominant determinants on microparticles, exosomes, and HIV-1 virions that arise by budding through the membranes of stressed host cells.

B cell receptor encoded by V_H_4-34 rearrangements recognize I/i determinants via contact sites associated with a V_H_ germline framework subdomain sequences – there is little apparent contribution from the somatically generated heavy chain CDR3 or by the paired light chain (Pascual et al., 1991). Using the 9G4 anti-idiotype antibody, B cells that bear non-mutated V_H_4-34 products have been shown to be highly represented, and whereas in healthy individuals these autoreactive germline B cells were shown to be excluded from T cell-dependent germinal center reactions (Pugh-Bernard et al., 2001), these V_H_ defined clones can be actively recruited into the germinal center reactions in patients with systemic lupus erythematosus (SLE; Cappione et al., 2005). These findings have been interpreted as evidence of immune defects in SLE patients related to the regulation of autoreactive B cells, although this topic remains controversial.

## NATURAL ANTIBODIES AND IMMUNE RECOGNITION OF DAMAGED AND APOPTOTIC CELLS

During the process of AC death, different cell membrane-associated phospholipids can undergo selective enzyme-mediated and oxidation associated modifications, and these cell membrane neo-determinants become available for recognition by the immune system. Among these, phosphatidylserine (PS) becomes oxidized and rapidly translocates from the inner to the outer leaflet of the cell membrane upon the initiation of apoptosis, where it can serve as a recognition signal for ingestion by professional phagocytes (i.e., “eat me” signal). Apoptosis can also be associated with other lipid neo-determinants, such as malondialdehyde (MDA), which is formed from interactions of unsaturated lipids with reactive oxidation species. Oxidative modifications of the abundantly distributed neutral phospholipid, PtC, also affect the distribution and/or conformation of the PC head group (Friedman et al., 2002), which renders it accessible for antibody recognition. These PC-antigens, as well as MDA-containing antigens, are immunodominant within murine B cell clonal responses that are boosted by intravenous infusions of ACs (Chen et al., 2009b).

The dominant natural antibody-producing anti-PC B cell clone, termed T15 (also TEPC15), has recurrently been isolated in anti-PC responses. The high representation of this clone in the early repertoire in part reflects a bias for increased representation of the specific V_H_S107.1 gene rearrangements used by the T15 clone (Feeney, 1991). In fact both of the V_H_ and V_L_ rearrangements in the T15 clone are formed by primary sequence direct rearrangements, and are without somatic mutations. The invariance of the T15 clonotypic NAb therefore is reminiscent of germline encoded receptors of the innate immune system (discussed in Shaw et al., 2000). In fact, T15 clonotypic antibodies are highly specific for PC determinants (Kearney et al., 1981) and in microarray analysis demonstrated little or no cross-reactivity to a large number of structurally distinct antigens (Chen et al., 2009b). Throughout life, T15-related B-1 cells are a major source of NAbs to a range of PC-containing antigens (Masmoudi et al., 1990), including those present on AC membranes, oxidized low-density lipoprotein (LDL), as well as in pneumococcal bacterial cell wall polysaccharide (Shaw et al., 2003; Chou et al., 2009). Many other B-1 cell clones have been demonstrated to be polyreactive and relatively low-affinity (Kantor and Herzenberg, 1993). However, crystallographic analysis of a V_H_S107.1 encoded (i.e., T15-related) Fab revealed a deep antigen-binding cleft with substantial binding affinity for the small PC moiety (reviewed in Davies et al., 1975). As a consequence of the dependence of the *in vivo* anti-PC response on T15 clonotypic B cells, otherwise immunocompetent mice which were made deficient only for the S107.1 V_H_ gene segment, have highly impaired responses to immune challenge with PC determinants on either ACs or bacteria, and also display impaired immune defenses for *S. pneumoniae* infection (Mi et al., 2000; Chen et al., 2009b). Taken together, these studies suggest that the antigen binding sites of T15-related antibodies have innate-like properties for recognition of PC-containing antigens are highly represented in the pre-immune repertoire (Kearney, 2005), in part because of their preferentially formation by biases in the somatic diversification mechanisms (Feeney, 1992).

Within the NAb pool there are also other antibodies that recognize distinct sets of neo-determinants that arise following cellular injury. There are at least two self-antigen specificities that have been reported to be associated with post-ischemic injury of endothelial cells (Zhang et al., 2008; Kulik et al., 2009). In addition, there are IgM–NAbs that specifically recognize erythrocytes with cell membrane-associated changes due to senescence or from damage by experimental treatment with the protease, bromalein (Cox and Hardy, 1985; Mercolino et al., 1986; Hardy and Hayakawa, 2005). These antibodies are reported to recognize a determinant involving PtC, although it is unclear whether the accessibility of this PtC-associated red cell epitope results from oxidative modification, or due to loss of erythrocyte membrane proteins. Red cell membrane intrinsic proteins have also been implicated as antigenic targets for IgG–NAbs (Lutz, 2012). Notably, as red cells have neither mitochondria nor nuclei, these cells do not undergo the same apoptosis-associated metabolic changes seen in conventional mitochondria-containing cells. This may explain why PC-related antigens are not prominently displayed on erythrocytes as a consequence of aging. Taken together, the cumulative data suggest that the IgM repertoire may include a range of distinct subsets of autoreactive NAbs, which recognize different cell types affected by apoptosis, injury and senescence, and these NAbs may help to regulate the clearance of different cell types and tissue remodeling as well as modulate innate immune responses.

## EFFECTS OF PC-SPECIFIC NATURAL IgM ON TOLL-LIKE RECEPTOR-INDUCED INFLAMMATION

Earlier studies have shown that C1q can directly binding to AC membranes and then serve as an “eat-me” signal for the phagocytic clearance of these dying cells (Korb and Ahearn, 1997; Navratil et al., 2001; Ogden et al., 2001). In explanation, C1q may directly interact with externalized PS on these damaged cells (Paidassi et al., 2008). In some settings, the deposition of C1q onto ACs can subsequently have an immunomodulatory effect and inhibit the secretion of proinflammatory cytokines, although by itself these effects are limited (Fraser et al., 2009). Similar properties have also been associated with the mannose-binding lectin (MBL), which triggers the lectin pathway of complement activation. MBL is structurally related to C1q and these two recognition molecules share a common ancestral genetic origin (Matsushita et al., 2004). Furthermore, MBL can also bind directly to ACs. This may suggest that initiation of apoptosis is associated with a change in the distribution of high-mannose glycoconjugates on the cell membrane (Stuart et al., 2005). These findings are consistent with reports that phagocytes of C1q-deficient mice, as well as MBL-deficient mice, display defects in AC clearance (Quartier et al., 2005; Stuart et al., 2005).

**FIGURE 1:**
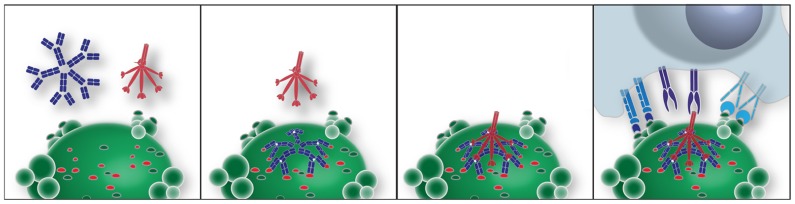
**Model of an IgM–NAb complex that enhances interactions between an apoptotic cell and professional phagocytes.** In this idealized model, apoptotic death results in membrane alterations that expose a range of neo-determinants. PC-associated membrane determinants are recognized by antigen-binding sites of a pentameric IgM. Binding of PC determinants results in conformational changes in the mu constant regions, which expose a conformational site responsible for recruitment of the globular heads of C1q (Czajkowsky and Shao, 2009). Alternatively, MBL binds to nearby high mannose N-linked glycoconjugates on mu-associated sites (not shown). This polymeric IgM–C1q complex is involved in generating or stabilizing interactions with receptors on professional phagocytes, which enhance apoptotic phagocytosis and blocks inflammatory responses.

The potential roles of T15 IgM–NAb have been investigated in the innate immune responses of professional phagocytes. As mentioned above, while this IgM natural antibody does not bind healthy cells it can specifically recognize exposed PC determinants on ACs and form complexes (Chen et al., 2009a,b). In turn, these AC–IgM complexes have greatly enhanced capacity to recruit the early complement factors, C1q and the structurally related MBL, at levels several-fold higher than in the absence of bound IgM. Notably the recruitment of C1q or MBL by IgM–NAb complexes greatly amplifies the capacity for AC phagocytic clearance (Chen et al., 2009a,b; illustrated in **Figure 1**). These properties are explained by reports that some polymeric IgM, when bound to their cognate antigen, are highly efficient at recruitment of C1q, while other studies have shown that polymeric IgM themselves can contain high mannose glycoconjugates (Arnold et al., 2006). Hence, AC-reactive polymeric IgM may serve to integrate these complement associated innate immune functions (Quartier et al., 2005; Chen et al., 2009a,b).

The formation of IgM–NAb complexes with ACs can also result in strong suppression of *in vivo* and *in vitro* inflammatory responses, including those induced by ligands for both membrane-associated and endosomal Toll-like receptors (TLRs), which include TLR3, TLR4, TLR7, and TLR9 (Chen et al., 2009b). These activities are also dependent on the recruitment of C1q and MBL, which are postulated to serve as bridging molecules that trigger phagocyte functions in a way that does not require activation of the complement cascade (Chen et al., 2009a,b). Hence, both the enhancement of apoptotic clearance and the down-modulation of inflammatory responses are therefore pathways by which some NAbs may augment and amplify housekeeping functions that serve to protect the host.

## EFFECTS OF THE APOPTOTIC CELL-SPECIFIC NAb–IgM ON IMMUNE COMPLEX DRIVEN PATHOGENESIS

During autoimmune pathogenesis high-affinity IgG autoantibodies can make direct contributions by multiple mechanisms (reviewed in Elkon and Casali, 2008; Lleo et al., 2010). Central to these pathways, many IgG autoantibodies implicated in systemic autoimmune diseases form immune complexes (ICs) with their target antigen, which alter their potential interactions with the host immune system. In sera of patients with conditions such as SLE and Sjogren’s syndrome, nucleic acid-recognizing autoantibodies can form ICs with their antigens, ribonucleoproteins, or deoxyribonucleoproteins. These ICs can deposit in tissues such as the kidney, skin, and joints and drive local inflammation and tissue injury by triggering complement cascades (reviewed in Bagavant and Fu, 2009). IgG-ICs might additionally serve as a delivery system to transport self-antigens to endosomal pattern-recognition receptors (PRRs) via uptake by activating Fc receptors. Studies by Marshak-Rothstein, Rifkin, and Rönnblom have demonstrated that DNA or RNA-containing ICs consisting of IgG autoantibodies bound to nuclear debris from dying cells can activate mouse B cells (Leadbetter et al., 2002), conventional dendritic cells (DCs; Boulé et al., 2004), and plasmacytoid DCs (Yasuda et al., 2007) as well as human peripheral blood mononuclear cells (Lövgren et al., 2004). These IgG-nucleic acid ICs can interact with rheumatoid factor autoantibody-bearing B cells (Leadbetter et al., 2002) or with DCs bearing activating FcγR, which enables the delivery to otherwise inaccessible intracellular compartments. The outcome of this targeted DNA/RNA internalization is the activation of PRRs of the innate immune system, such as the nucleic acid-recognizing TLRs; TLR7 (activated by ssRNA) and TLR8 and TLR9 (activated by DNA). IgG antibodies to the citrullinated self-protein, fibrinogen, which are prevalent in rheumatoid arthritis patients, have also been shown *in vitro* to activate macrophages through a co-stimulatory pathway involving both FcγR and TLR4 (Sokolove et al., 2011).

The pathogenic influence of IgG autoantibody-ICs can be opposed by IgM natural antibodies to ACs. *In vivo* studies have shown that administration of anti-PC natural IgM greatly attenuated disease severity in a murine model of collagen-induced arthritis (CIA; Chen et al., 2009a). In this model, immunization with xenogenic collagen II emulsified in complete Freund’s adjuvant induces a pathogenic autoimmune response to type II collagen (Terato et al., 1992; Nandakumar et al., 2003), with tissue injury in part mediated through the activating Fc-γ receptors (Kleinau et al., 2000). Infusions of IgM–NAbs to ACs also blocked the disease process induced by passive transfer of anti-type II collagen autoantibodies (Chen et al., 2009a), in which inflammatory arthritis is mediated by FcγR and innate immune cells, while lymphocytes do not play central roles (Terato et al., 1992; Nandakumar et al., 2003).

*In vitro* studies have shown that anti-AC IgM antibodies can directly block the activating effects of lupus-associated IgG autoantibodies on bone marrow-derived DCs (Vas et al., 2012). In fact, the inflammatory effects of both anti-DNA and -RNA IgG-nucleic acid ICs in myeloid DCs were inhibited with suppression of the secretion of inflammatory cytokines IL-6 and TNF-α (Vas et al., 2012). This IgM–NAb also suppressed IC-mediated induction of cell surface expression of CD80 and CD86, as well as CD40 and other co-stimulatory molecules.

## NATURAL ANTIBODY REGULATORY PATHWAYS THAT MODULATE INFLAMMATORY AND AUTOIMMUNE DISEASES

Serologic surveys of a large cohort of well-characterized SLE patients have further evaluated the potential clinical relevance of IgM autoantibodies to defined oxidation-associated antigenic-specificities, including the apoptosis-associated neo-antigens, PC and MDA. In the lupus cohort, levels of both of these types of IgM autoantibodies were significantly higher compared to healthy adult controls (Grönwall et al., 2012a). Importantly, higher levels of IgM anti-PC correlated with less long-term organ damage, as defined by the SLICC/ACR damage index score, as well as lower disease activity as assessed by the SELENA revision of the SLE disease activity index (SLEDAI) at the time of visit. IgM anti-PC levels also correlated with an absence of cardiovascular events, while there were no associations with renal disease (Grönwall et al., 2012a). These results are consistent with a previous report from a smaller cohort of Swedish patients (Su et al., 2008) and with studies showing that lower IgM anti-PC levels are associated with more frequent cardiovascular events in non-autoimmune patients (de Faire et al., 2010; Fiskesund et al., 2010). These findings were in fact predicted by earlier studies in atherosclerosis-prone mice (Shaw et al., 2000). Indeed, pneumococcal vaccination, which induces PC-specific antibody responses, was shown to arrest plaque progression in LDL receptor-deficient mice with cholesterol levels over 1000 mg/dl (Binder et al., 2003). These findings have therefore further strengthened the hypothesis that some anti-AC IgM–NAbs can play protective roles in inflammatory disease.

Yet not every IgM–NAb that recognizes ACs may have equivalent clinical implications. In fact, levels of antibodies to PC and to MDA showed significant differences in their associations with lupus clinical manifestations. IgM anti-MDA showed only weak inverse correlations with the SLICC/ACR damage index but not the SELENA-SLEDAI score and there were also no significant associations with renal disease or cardiovascular events (Grönwall et al., 2012a). These studies also showed that higher levels of the IgM antibody to β2-GPI correlated with less organ damage by SLICC/ACR damage index. Furthermore, patients without renal disease had higher levels of IgM anti-CL, and IgM anti-dsDNA (Grönwall et al., 2012a). This may indicate that higher levels of some IgM antibodies may protect some patients from kidney disease, as suggested in an earlier and more focused report (Mehrani and Petri, 2011). Taken together, these studies refute the notion that circulating IgM autoantibodies are inherently polyreactive. Instead, these data strongly argue that the antigenic fine binding specificity of the IgM determines whether there is an association with protection from certain lupus disease features (Grönwall et al., 2012a). In a recent clinical study, Ajeganova et al. (2011) examined RA patients treated with TNF-α blockers. They observed that levels of PC-specific natural IgM levels were increased in patients treated with TNF-α blockade, while lower anti-PC IgM levels correlated with inferior response to therapeutic intervention for RA disease. Further investigations will be needed to better understand how anti-AC NAbs may modulate the pathogenesis of different autoimmune rheumatic diseases.

## MAPK PHOSPHATASE-1 IS REQUIRED FOR NATURAL ANTIBODY SUPPRESSION OF TLR RESPONSES

Investigations of signal transduction pathways have shown that IgM–NAbs to ACs can affect responses induced by agonists for a broad range of TLRs, by inhibition of the mitogen-activated protein kinase (MAPK) signal transduction system, which plays central roles in the induction and resolution of inflammatory responses (Grönwall et al., 2012b). Inflammatory responses can result from the induction of phosphorylation of one or more of the primary MAPKs; ERK1/2, JNK, and particularly p38, which then translocate to the nucleus where it can affect transcriptional regulation. In rheumatoid arthritis, activated (phosphorylated) p38 is increased in the RA synovium. However, despite evidence that small molecule p38 inhibitors have been effective in mouse models of inflammatory arthritis, efficacy in humans has been limited, which has suggested that an alternate approach to MAPK inhibition may provide greater clinical benefits (reviewed in Hammaker and Firestein, 2010).

To assess the potential relevance of this type of immunomodulatory NAb to clinical autoimmune diseases, the activity of the PC-specific IgM–NAb was also tested in a system in which inflammatory responses are induced by lupus IgG autoantibodies (Vas et al., 2012). These studies demonstrated that this natural IgM inhibited p38 phosphorylation induced in DCs by nucleic acid-containing IgG autoantibody ICs (Vas et al., 2012). Notably, this inhibitory pathway also blocked the nuclear accumulation of the activated primary MAPKs in myeloid DCs (Vas et al., 2012). *In vitro* studies of murine bone marrow-derived DCs confirmed that this inhibition was entirely dependent on the recruitment of either C1q or MBL (Grönwall et al., 2012b; illustrated in **Figure 1**).

The magnitude and duration of MAPK signaling is dependent on the balance between the upstream activators of the system and the deactivation of these kinases by specific phosphatases. Based on evidence that this NAb could affect the activation of each of the three primary MAPKs (Grönwall et al., 2012b), studies were therefore performed that assessed the potential involvement of the regulatory MAPK phosphatases (MKPs), also known as dual-specificity phosphatases (DUSPs). These studies highlighted the role of MKP-1, the archetype for the family, which can serve as the counter-regulatory factor for all three of the primary MAPKs (reviewed in Liu et al., 2007). In fact, the anti-AC IgM-mediated blockade of TLR-mediated MAPK signaling had an absolute requirement for the expression of MKP-1 (Grönwall et al., 2012b). In DCs activated by TLR agonists, the addition of the anti-AC IgM, in the presence of C1q or MBL in serum-free media, resulted in induction within minutes of MKP-1 at a transcript and a protein level, and it rapidly became localized within the nucleus (Grönwall et al., 2012b). Using deconvolutional immunofluorescence microscopy, NAb-mediated MKP-1 accumulation correlated with a reciprocal impairment in the phosphorylation and nuclear translocation of the activated primary MAPK protein molecules. To investigate the relative contribution of MKP-1 to NAb-mediated suppression, responses were compared in DCs from wild-type or MKP-1-deficient mice (Grönwall et al., 2012b). Such MKP-1-deficient mice are reported to exhibit overexuberant inflammatory responses, but no other immune developmental abnormalities (Dorfman et al., 1996; Salojin et al., 2006). These studies confirmed the absolute requirement for MKP-1 for IgM–NAb-mediated inhibition of TLR responses from DCs (Grönwall et al., 2012b).

## CONCLUDING REMARKS

One of the most fundamental challenges faced by the immune system is the efficient recognition and clearance of the body’s own cells, which because of senescence or injury enter programmed cell death pathways. While cells dying of apoptotic death pathways do not pose an immediate risk to the host, if these cell corpses are not efficiently removed there is the risk of progression to secondary necrosis. This can lead to the loss of integrity of cell membranes with release of cytoplasmic and nuclear components that can serve as ligands for proinflammatory cellular receptors, and the triggering of autoimmune responses. Hence, throughout the lifespan of multicellular organisms, there is an absolute need for the clearance of the immense number of cell corpses that are generated each day, even in health. As a direct consequence, the immune system has developed a redundant layering of superimposed mechanisms. Hence, the control of apoptotic clearance is intertwined with the regulation and resolution of inflammatory responses.

At birth, humans already have substantial levels of circulating IgM antibodies, which reflect a functional B cell compartment poised and ready to contribute to neonatal host defenses. These IgM antibodies arise in the womb from neonatal B lymphocytes that express clonally distributed BCRs. However, evidence of recurrent clones suggests that this early B cell repertoire may be affected by *in vivo* clonal selection that may be a response to evolutionary pressure to provide important housekeeping functions related to apoptotic clearance and avoidance of excessive and damaging inflammatory responses.

IgM-mediated protection from autoimmune disease was first demonstrated in mice deficient in the capacity to secrete IgM antibodies, as these mice were found to develop pathologic autoimmunity with the production of lupus IgG autoantibodies (Boes et al., 2000; Ehrenstein et al., 2000). Furthermore, in mice predisposed to the development of lupus-like disease, a bias toward secretion of monomeric IgM and lower levels of polymeric IgM can result in accelerated development of lupus-like disease (Youd et al., 2004). It may therefore be relevant that 8% of a cohort of 300 SLE patients were recently reported to have selective deficiency in serum IgM (Perrazio et al., 2012).

Our studies demonstrated that anti-AC IgM–NAbs, present from early in life, can suppress inflammatory responses mediated by phagocytic cells by induction of MKP-1, which in other settings have been shown to have potent regulatory roles for the MAPK system. MKP-1 is well known for its many counter-regulatory roles, which include the late negative feedback of responses to LPS stimulation, the blunting of responses after rapid re-exposure to a TLR agonist such as LPS tolerance, as well as contributing to the anti-inflammatory properties of glucocorticoids (reviewed in Liu et al., 2007). These studies also documented an additive effect of anti-AC IgM–NAb and dexamethasone for early MKP-1 induction and inhibition of LPS-induced p38 MAPK activation that, when combined, exceeded the maximum effects of either agent alone (Grönwall et al., 2012b). In part, this is likely explained by the additive integration of separate signals received via distinct cell membrane-associated receptors triggered by dexamethasone (i.e., glucocorticoid receptor) or by anti-AC IgM complexes (discussed below). As glucocorticoids are amongst the most widely prescribed treatments for inflammatory and autoimmune diseases, it is indeed intriguing that there is an overlap in the inhibitory signal transduction pathways of glucocorticoids and by the formation of regulatory ICs with early complement recognition factors that are coordinated in their organization by IgM autoantibodies to oxidation-associated neo-determinants on ACs.

Fundamental to the inhibitory effects of regulatory NAbs, polymeric IgMs that bind ACs can express constant regions with multiple sites for recruitment of C1q, and the Fcμ of some IgM–NAbs also have high mannose glycoconjugates on that bind MBL (Chen et al., 2009a,b). The potential properties of such complexes suggested by studies with targeted deficiencies in C1q, MBL, or secreted IgM, which each have impaired control of inflammatory responses, and in some cases are predisposed to the development of autoimmune disease (Botto et al., 1998; Boes et al., 2000; Ehrenstein et al., 2000; Stuart et al., 2005). Furthermore, we have previously shown that the complement-dependent immunomodulatory properties of anti-AC IgM, while the recruitment of C1q or MBL was essential, there was no absolute requirement for downstream activation of the complement cascade (Chen et al., 2009a).

These IgM–NAbs to AC-associated determinants can regulate responses mediated by diverse TLRs, an ancient type of innate immune receptor that was first characterized in insects (Lemaitre et al., 1996). Furthermore, mechanistic investigations have shown these effects are linked to modulation of the MAPK signaling system, which is one of the earliest evolutionarily conserved pathways of immunity, being present in plants and mammals (Asai et al., 2002). Likewise, MKP-1 orthologs have also been described in protozoans (Moncho-Amor et al., 2011), and as mentioned above, mice with MKP-1 deficiency have severe defects in the control of innate responses (Salojin et al., 2006). These regulatory properties are expressed by a class of naturally occurring autoreactive antibodies that are postulated to come from the most primitive tier of B cells in the adaptive immune system (Kantor and Herzenberg, 1993).

As ACs are ubiquitous, we wonder whether the high frequency of these innate-like NAb-producing clones in the “preimmune” repertoire in part reflects positive selection of the B-1 cell clones that are reactive with membrane-associated neo-determinants on cells wasted during development. The protective properties of anti-AC NAbs may be mediated by a previously unknown regulatory signaling pathway, which integrates and coordinates the influence of select innate immune factors on myeloid cell function.

## Conflict of Interest Statement

The authors declare that the research was conducted in the absence of any commercial or financial relationships that could be construed as a potential conflict of interest.
